# Postpartum Health Information Seeking Using Mobile Phones: Experiences of Low-Income Mothers

**DOI:** 10.1007/s10995-016-2185-8

**Published:** 2016-09-17

**Authors:** Lucia Guerra-Reyes, Vanessa M. Christie, Annu Prabhakar, Asia L. Harris, Katie A. Siek

**Affiliations:** 1 Department of Applied Health Science, School of Public Health, Indiana University Bloomington, 1025 E 7th Street, Suite 116, Bloomington, IN 47405 USA; 2 Department of Informatics, School of Informatics and Computing, Indiana University Bloomington, 919 E. 10th Street, Bloomington, IN 47408-3912 USA; 3 Department of Epidemiology, School of Public Health, Indiana University Bloomington, 1025 E 7th Street, Suite C028, Bloomington, IN 47405 USA

**Keywords:** Postpartum, Mobile phones, Internet, Low-income women, Health IT

## Abstract

*Objectives* To assess low-income mothers’ perceptions of their postpartum information needs; describe their information seeking behavior; explore their use of mobile technology to address those needs; and to contribute to the sparse literature on postpartum health and wellness. *Methods* Exploratory community-based qualitative approach. Interviewees were recruited among clients of community partners and had children aged 48 months and under. A survey assessing demographics was used to identify low-income mothers. 10 low-income mothers were recruited from survey participants to complete in-depth interviews regarding postpartum information needs, information seeking, and technology use. Interviews were transcribed verbatim and coded by three researchers independently. Narratives were analyzed along predetermined (etic) and emergent (emic) categories. *Results* Establishing breastfeeding and solving breastfeeding problems were central postpartum concerns leading to information seeking. Interviewees reported almost exclusive use of mobile phones to access the Internet. Mobile applications were widely used during pregnancy, but were not valuable postpartum. Face-to-face information from medical professionals was found to be repetitive. Online information seeking was mediated by default mobile phone search engines, and occurred over short, fragmented time periods. College graduates reported searching for authoritative knowledge sources; non-graduates preferred forums. *Conclusions for Practice* Low-income postpartum women rely on their smartphones to find online infant care and self-care health information. Websites replace pregnancy-related mobile applications and complement face-to-face information. Changes in searching behavior and multitasking mean information must be easily accessible and readily understood. Knowledge of page-rank systems and use of current and emergent social media will allow health-related organizations to better engage with low-income mothers online and promote evidence-based information.

## Significance

*What is already known?* New mothers increasingly use the Internet to find social support and information on self-care and infant care. Individuals with a lower household income use the Internet less than higher income groups (Perrin and Duggan 2015). Low-income women have been reported as less likely to use online sources of health information during pregnancy and more likely to use interpersonal sources of information (Song et al. 2013). *What this study adds?* Online sources are fundamental for postpartum low-income women, who seek information and reassurance online. Smartphone search engines are their main information gateway. A pattern of short bursts of searching while multitasking means information must be easily accessible and readily understood. Organizations seeking to provide low-income mothers with evidence-based information need to understand page-rank systems and use multiple streams of information, including current and emergent social media.

## Introduction

Adequate care and support during the postpartum period have been linked to positive health behaviors, and health outcomes in high risk populations such as low-income mothers. Access to adequate health information in the postpartum period can reduce maternal stress and promote infant health (Shaw et al. 2006). During the transition period of adjusting to life with a newborn, the need for health information increases as the available time to search for it decreases (George 2005). During pregnancy, women utilize a variety of sources of health information, including medical providers, books and magazines, friends and family, and a multitude of online sites and mobile applications. Many of these sources, however, do not extend significantly into the postpartum period and to infant care.

Postpartum women find themselves in the contradictory situation of having received an abundance of information, yet feeling that they know very little about how to manage the day-to-day needs of life with a newborn (George 2005; Cheng et al. 2006). Women often report that prenatal education prepared them for labor and delivery, but not for the postpartum period and the realities of self-care and infant care (McVeigh 1997; Moran et al. 1997; Sword and Watt 2005). As the emphasis of healthcare switches markedly to the needs of the child (Kapp 1998), women experience an overwhelming responsibility. Often, new mothers feel inadequately prepared to care for their infant, leading to feelings of stress, isolation, and frustration (George 2005; Cheng et al. 2006; Kanotra et al. 2007).

Online information seeking during pregnancy has been widely reported (Larsson 2009; Shieh et al. 2010; Lowe et al. 2009; Lima-Pereira et al. 2012; Rodger et al. 2013). Research on information needs and information seeking behaviors in the postpartum period has been limited, however. One study of postpartum information seeking reported a high prevalence of online searches following childbirth (Bernhardt and Felter 2004). Additionally, online social networking platforms like Facebook have become important sources of information, advice seeking, and social support for mothers (Morris 2014).

Low-income women have a greater need for health information (Sword and Watt 2005; Landy et al. 2008), yet do not rely on Internet sources as much as other interpersonal sources (Song et al. 2013). Limited access to the Internet among low-income families during the early 2000s may provide an explanation for this disparity (Zickuhr and Smith 2012). The recent expansion of web-enabled smartphones has increased access to the Internet in the U.S., with 64 % of Americans reporting smartphone ownership in 2015 (Smith 2015). Low-income smartphone owners are more likely to rely on their smartphones as a primary source of Internet access (Smith 2015; McHenry 2016).

Recent studies show that postpartum women now use information and communication technologies to search for health information more frequently (Gibson and Hanson 2013; Jang et al. 2015). However, we do not know if increased access to the Internet has affected information seeking for postpartum low-income mothers. The objectives of this study were to explore early postpartum information needs of low-income mothers, describe their information seeking behaviors, and assess their use of mobile technology to fulfill their information needs.

## Methods

We formed a community-based research partnership with the Monroe County Women, Infants and Children (WIC), and Bloomington Area Birth Services (BABS), a community-based nonprofit that promotes maternal and child health. An exploratory, sequential, quantitative–qualitative design was used to address the research objectives. This methodological approach is recommended to identify and access marginal members of a population such as low-income households (Creswell 2014). A convenience sample survey served as a method for identifying low-income mothers. Qualitative in-depth interviews were conducted to explore the experiences of low-income women specifically. Here, we report on the qualitative results of the study. This study was approved by the Indiana University Institutional Review Board. All participants provided consent for all data collection methods and recordings.

Survey participants were recruited among clients of community partners as part of a research agreement. Participants were mothers residing in Monroe County with at least one biological child aged 48 months or younger. A total of 82 surveys were initiated, and 77 surveys were completed. 33 survey respondents were low-income mothers, identified by Medicaid participation^1^ (http://www.Medicaid.gov).

Interviewees were recruited from low-income survey respondents who indicated a willingness to participate in further research (n = 23). All 23 women were contacted, 12 responded to initial and follow-up contact, and 10 women completed the interview. Interviewers were female, identified themselves as researchers and, in one case, a fellow mother of young children. Interviews were conducted in the interviewees’ chosen place—most often their homes. Semi-structured interviews focused on broad issues: experiences of the early postpartum period; information needs (pre and postpartum); sources of information; and technology use. Questions were open-ended (i.e., tell me about your most pressing questions after [child] was born?) to allow interviewees to tell their stories (Denzin and Lincoln 2011).

Each interview was recorded and transcribed verbatim. The content of each interview was read and analyzed by three members of the research team. Predetermined (etic) categories directly related to interview areas, and emergent (emic) categories were used for content analysis (Hsieh and Shannon 2005). The lead author, a trained qualitative researcher, led weekly meetings to analyze coding patterns, resolve conflicts, identify emergent themes, align codes, and ensure consistency throughout analysis. Analysis was aided by the collaborative cloud-based software Dedoose (http://www.Dedoose.com 2014).

## Results

### Interviewee Demographics

A total of 10 low-income women participated in the qualitative interviews. Interviewee demographics are listed in Table 1.

**Table 1:** Interviewee demographics (N = 10)

	N (%)
**Age** (mean ± SD)	29.9 ± 3.6
**Race**	
Asian	1 (10)
White	8 (80)
More than one Race	1 (10)
**Ethnicity**	
Hispanic or Latino	2 (20)
Not Hispanic or Latino	7 (70)
Not reported	1 (10)
**Education level**	
High school diploma	2 (20)
Some college	2 (20)
Associate’s degree	1 (10)
Bachelor’s degree	4 (40)
Doctoral degree	1 (10)
**Income**	
$0–$15,000	2 (20)
$15,001–$30,000	4 (40)
Not reported	4 (40)
**Marital status**	
Living with partner	1 (10)
Married	7 (70)
Divorced	1 (10)
Separated	1 (10)
**Size of household** (including self)	
2	1 (10)
3	4 (40)
4	4 (40)
5	1 (10)
**Number of children**	
1	5 (50)
2	4 (40)
3	1 (10)
**Number of people cared for** (excluding self)	
1	2 (20)
2	5 (50)
3	3 (30)
**Services utilized**	
WIC	9 (90)
Bloomington area births services	7 (70)
Medicaid	8 (80)
Mobile phone type	
Smart phone	8 (80)
Feature phone	2 (20)

### Information Needs

Interviewees expressed four areas of information needs that led to information seeking in the early postpartum: establishing breastfeeding, breastfeeding problems (tongue tie and lip tie); general health and behavioral issues; and topics that some interviewees found difficult or uncomfortable to discuss with healthcare providers (Table 2).

**Table 2:** Salient postpartum information needs

Information topic (N interviewees who identified topics as information needs/total N)
**Establishing breastfeeding **(10/10)
“I had a really hard time breastfeeding. I would cry every time he needed to eat, I was in so much pain. It was a painful and emotional transition…I remember being afraid to call BABS or look for someone to help me breastfeed. I don’t know exactly why I felt that way, but I felt more isolated. I do remember talking to my grandma and my aunt about breastfeeding and the pain that I was going through with breastfeeding. My grandma said she didn’t breastfeed and my aunt said she breastfed for a few weeks, six weeks or something.” (P2)
“My biggest questions were about breastfeeding, I didn’t breastfeed my boys because I was young and I didn’t know any better. But when I had her …I knew I wanted to do it, but we ended up having a lot of issues with her latching and stuff, so I needed a lot of help with knowing just about latching and breastfeeding in general.” (P6)
**Tongue tie and lip tie **(4/10)
“I felt like after my first I had book smarts, I’d read things I’d talked to people. And then it all happened. He was tongue tied and lip tied which my first one wasn’t. And so weeding though the information on that and finding him help for that was a big thing in our first couple of weeks together.” (P10)
“She couldn’t latch well and we later found out it was a tongue tie. I was like freaking out that something was wrong with her, because I was having so much pain. So I was like Googling and researching like what are different things that could be going on, why is breastfeeding hurting? It didn’t hurt last time.” (P5)
**Infant’s health issues** (6/10)
“I had a lot of issues with like growth and development, like things would be going one way for a little while and then all of a sudden she wouldn’t have an appetite or she would stop sleeping or she would be cranky all the time. So a lot of it was like going online and being like is she sick, what is going on, is she okay?” (P1)
“I looked up about his eye being gunky like that… and researched the right way to massage that, because you can massage his face to try to get that [tear] duct to open up.” (P9)
**Topics described as difficult or uncomfortable to discuss with healthcare providers** (5/10)
“Yeah, sex after baby that is definitely a good one. That was uncomfortable, I don’t think I ever did ask, because I was uncomfortable asking. I think they just brought it up to me and stuff.”(P6)
“I think I looked at something about depression. That I never talk about with others, but I do not have concerns about myself, because I think I am not depressed. I think I looked for the information just to understand.”(P8)

Overall, breastfeeding was the most pressing issue that led to information seeking in the postpartum period. Interviewees were committed to breastfeeding, but several found the process difficult to establish. Struggles with breastfeeding highlighted the differences between children and the lack of experience-based knowledge of breastfeeding within interviewees’ immediate families (Table 2). Another salient issue for interviewees was breastfeeding problems like tongue tie and lip tie, which led even experienced mothers to seek information and guidance (Table 2). Often, online searches were prompted by a desire for reassurance about a child’s health-related issues. Some interviewees referenced searching for topics that they felt were difficult or uncomfortable to discuss with healthcare providers, such as postpartum intimacy and depression.

### Navigating Information Streams

Interviewees utilized multiple information streams (Table 3) to search for postpartum health issues—from in-person medical professionals to authoritative online sources to Internet forums. Interviewees transitioned through several information streams in search of answers:

**Table 3:** Sources of postpartum information mentioned by interviewees

Sources Mentioned	Face to face or phone	Printed materials	Website	Expert forum	Parent forum	Social media	Mobile app	Type of postpartum concern for which source was used	Number of mentions by education level completed		
									College or higher	Less than college	Total
Kelly mom (breastfeeding resources by IBCLCs)			✓				✓	Breastfeeding	1	0	1
Leaky boob (a collection of articles on breastfeeding support and child nutrition)			✓			✓		Breastfeeding	1	0	1
Baby center (website providing information on conception, pregnancy, birth, and parenting)			✓	✓	✓		✓	Breastfeeding, child’s health, mother’s health	3	5	8
The bump (provides information on conception, pregnancy, birth, and parenting. Explicitly focused on millennial parents.)			✓				✓	Breastfeeding	1	0	1
Prego and mommy chat (Facebook community page for all mothers)						✓		Child’s health, breastfeeding	1	0	1
Ask Dr. Sears (website featuring advice from Drs. Sears on pregnancy, childbirth, and parenting)			✓					Child’s health, breastfeeding	1	0	1
CDC and NIH (Center for Disease Control/National Institute of Health)			✓					Child’s health, breastfeeding	1	0	1
Healthcare providers	✓	✓						Child’s health, breastfeeding, infant care	6	7	13
Friends and family	✓							Child’s health, breastfeeding, infant care	3	3	6
Community organizations	✓	✓						Breastfeeding	2	5	7
What to expect (information on pregnancy, birth and parenting early years)		✓	✓	✓	✓		✓	Breastfeeding	0	2	2
Parents magazine		✓	✓					Infant care	1	0	1
La Leche league (international breastfeeding advocacy group)	✓	✓	✓					Breastfeeding	1	0	1
NAEYC Facebook page (National Association for the Education of Young Children)						✓		Infant care	1	0	1
Facebook mom groups (non-specific)						✓		Infant care, breastfeeding	1	3	4
Mom-Blogs (non-specific)			✓					Infant care, breastfeeding	2	1	3
Magazines and books (non-specific)		✓						Infant care, breastfeeding, child’s health	1	2	3
Google result pages (non-specific)			✓					All of the above	0	3	3

- “I reached out to the pediatrician’s office and the lactation people there and they would give me handouts and things [for child’s latch]…*they [the handouts] would say the same thing…*There is a Facebook forum called Prego and Mommy Chat…. there are all these people who are responding [to questions]…these were just parents who have been through it and those seem to be some of the best sources for information.” (P5)
- “Sometimes if I want to know like a specific symptom or behavior specific, I will just Google. But other times, I will browse like Babycenter and we also have some website, medical websites in Chinese… I was also added to several parents groups on Facebook.” (P8)

These experiences highlight the difficulty mothers often have finding information postpartum and the sometimes repetitive, generalized information medical professionals provide. Websites represented an important source of information for interviewees. Internet searches were described as a more readily accessible and faster source of information than medical professionals. Books, family, and friends were also cited as sources of information. Additionally, Facebook was an important source of information and reassurance.

All interviewees looked for website recommendations from people or groups they trusted. However, some interviewees only searched for what they considered to be authoritative online sources for medical information and knowledge. For example:

- “[Leaky Boob on Facebook] It’s a [La Leche League recommended] evidence based hub of information for breastfeeding mamas. I try not to start just googling terms randomly. I’d rather have somebody that’s research based peer-reviewed. People that actually know what they are talking about.” (P10)

One participant specifically mentioned looking at accreditation and domain (e.g.,.org,.edu) “because I know that they have to have… some sort of filtering” (P5).

Others sought reassurance and social support through online forums, where members’ experiences, struggles, and solutions provided advice and assurance of normality:

- “I googled and would look through the links to find ones for websites that I was comfortable and familiar with, like Baby Center and What to Expect, and then I would find the ones that were expert advice or expert answers. I would look for those first and then I would do like the mom answers, the forum ones where people have said like this is going on or this is what I am experiencing… I would read through those as kind of a way to just feel connected to someone else just to know that I am not crazy…” (P5)
- “I punch in sentences and big questions in Google [as a] way of getting different experiences from different moms. Not so much the type of information, but knowing that other moms did this, knowing that it [tongue tie] was very common and not talked about pissed me off, because all these moms are dealing with this stuff. So yeah [I was drawn to] forum or like blogs of moms who have been there, like what they went through, their emotions” (P4)

We observed several differences in the types of sources mentioned by interviewee education level (Table 3). Women with a college degree mentioned the importance of finding sources of authoritative knowledge (i.e. professional organizations or academic sites) more frequently than those without a degree. Women without a college degree mentioned using forums like those available at BabyCenter as a form of reassurance, while seeking medical knowledge from their health providers and WIC.

### Accessing Information Streams

Most interviewees searched the Internet using their smartphones. They described how their phones were always with them, easy to access, and connected to the Internet:

- “Mostly my phone, because I don’t get a spot in front of the computer, since I stopped being pregnant, very often. So 90 % on my phone.” (P4)
- “I always use my phone. I don’t have anything else besides that, so that is what I use. If you do have a question while you are at the doctor’s office or something, you can look something up and show it to him.” (P6).

Searches occurred through the phone’s default search engine, which in most cases was Google, Bing, or Yahoo.

Despite the convenience of smartphone use for accessing data, time spent looking for information was described as very short:

- “Short bursts, it was more short bursts. Now when I was pregnant it was long, but after being pregnant, it was like became short bursts.” (P3)
- “Usually it is just 5 to 10 min if I can get that much time and sometimes whenever my kids are sleeping, sometimes I can spend a couple hours looking up stuff, but that is rare, because she doesn’t sleep without me.” (P6)
- “Like little snippets of time, so I would pull up the site or something and I would leave it up so when I had five minutes I could go read a little bit about it, and then if it was like after the kids had gone to bed at the end of the night, then okay now I can pour over this for however long I choose.” (P5)

Women multitasked while they used the Internet by watching television, nursing their child, or fulfilling other childcare responsibilities. Overall, interviewees described changing patterns in technology use before and after baby: from longer, continuous usage times that were dictated by their schedule to shorter, dispersed periods, the timing or duration of which they did not control.

### Mobile App Use and Issues in Pregnancy and Postpartum

Interviewees used diverse mobile phone apps during pregnancy and labor, but their use was reduced in the postpartum period. Interviewees using pregnancy apps would like to extend their use in the postpartum period. Understanding pregnancy app use, and women’s perceptions of their use illustrates important features of engaging with this population over mobile phones. The most mentioned pregnancy apps were The Bump, What to Expect and Text4baby. Interviewees mostly reported that app use during pregnancy was an enjoyable experience. However, some mentioned issues with what they perceived as the validity, applicability, and timing of the data presented by some apps:

- “Maybe some of the advice is outdated or it is not very complete. I guess I don’t have it that much. I am interested to see what it [The Bump app] says, but I don’t take it too very seriously.” (P2)
- “I think that some of the stuff in there that they try to say is normal may or may not be normal pertaining to each mother, but I kind of knew that by my third pregnancy….[since the information is] very wide range and it might make some moms feel uncomfortable.” (P6)
- “Yes, that [Text4baby] was cool, sort of, until it started giving the same information… It asks your due date and you are like okay you go forward. But they start repeating certain ones [text messages]. I know I freaked out when they started asking me about, ‘well do you want this at your labor?’ What the [expletive]! I just found out I am pregnant, can you let me adjust to this concept first!” (P4)

Interviewees also discussed instances when apps were not as helpful as they had hoped or they lacked the resources to use the apps:

- “I had apps trying to find something out and most of them were useless. Most of them were like stupid stuff, but you know just about everybody knows when you are pregnant you know you are probably going to have to pee more, stuff like that. I usually ended up erasing them because it was like okay this is like giving me stupid info.” (P4)
- “I had it [Babycenter app] for a little while, but it took up too much space on my phone, so I had to delete it.” (P9)
- “We had only a desktop but we didn’t have the greatest Internet connection out here [in rural Indiana]. We recently got a better Internet connection. Like [now] we have an iPod Touch which is like a smartphone, it is sort of more at my fingertips and I definitely use the Internet more.”(P7)

Resources like device constraints or inadequate Internet infrastructure were a common barrier to mobile app use during pregnancy. Furthermore, interviewees perceived that these apps did not extend their usefulness into the postpartum period.

A majority of interviewees reported using apps in the planning and pregnancy stages; however, app usage tapered off postpartum:

- “Not really—more so in the pregnancy stage, it was more so the ‘What to Expect’ app and post pregnancy it was more of the ‘BabyCenter’ app. They just focused on different things… the first two months really I used it [BabyCenter app] a lot more and then it just kind of tapered off.” (P1)
- “No, I haven’t found anything yet that helped [postpartum]… for a while I looked and there may have been one or two, but they were like expensive apps.” (P4)

The reduction in mobile app use postpartum may be due to a lack of available apps with postpartum content or a lack of awareness of the existence of postpartum apps. Accessibility may be another barrier to postpartum use of mobile apps by interviewees who found the apps to be too expensive. Both contribute to the lack of mobile app use in the postpartum. Women who previously used mobile apps, but did not find desired postpartum information in apps, often turned to social media or websites appearing in search engine results.

## Conclusions for Practice

Our results demonstrate that the Internet is the primary source of information for postpartum low-income women. Postpartum information needs focused on infant care, specifically breastfeeding, and managing feelings of inadequacy, stress, lack of experience, and lack of knowledge (Cheng et al. 2006; Sword and Watt 2005; Kanotra et al. 2007). In this context, online sources provide knowledge and reassurance, helping to normalize a stressful transition experience.

Smartphones are the main point of access to the Internet for low-income women. Many used mobile apps in pregnancy; however their usage did not extend into the postpartum period, creating a mobile app gap: that is in the perception of interviewees there were no free and useful apps for the postpartum. This gap was expressed in two ways: availability and quality. Fewer apps were available to mothers postpartum, either due to lack of awareness, or inability to afford existing ones. Secondly, women felt that existing postpartum apps were repetitive, non-validated, or contained irrelevant content. A possible explanation for this is that developers of mobile apps focus on preconceived notions of what women should know (Hayes et al. 2014), which may not reflect the actual information needs of new mothers.

Overall, websites were the main source of postpartum information. Our results suggest that preferred sources of information online varied by education level. College graduates gravitated toward specific websites linked to professional or academic organizations. In contrast, mothers of lower education levels gravitated toward peer-to-peer forum sites. This preference could be related to the perception of forums as a safe and freeing space (Schoenebeck 2013) or to lower levels of health literacy (Shieh et al. 2009). Notably, the differences observed here by education occurred among a small sample size in a study that is largely exploratory in nature. Regardless of education level, forums, blogs, and social media were increasingly mentioned as sources and hubs of information.

Changes in the method of information seeking from long periods of online searching during pregnancy to short, fragmented bursts of searching after the birth of a child conform to the unpredictable nature of time demands in the early postpartum period. A consequence of this search method is that information must be readily available and easily digestible to meet the time and attention constraints of the seeker. Recent studies also address other influences on fragmented, shortened periods of mobile phone use, such as pressure felt by parents to limit phone use while caring for their children (Hiniker et al. 2015).

The manner by which default search engines rank page results and social media and forum sites display content presents both opportunities and challenges for postpartum health education aimed at low-income mothers. Shared membership in a specific group may lend a higher level of trust to information circulated within the group. This can circulate non-evidence-based information, creating echo chambers where dissenting sources have little chance of appearing (Bakshy et al. 2015). However, participation in these groups is also a necessary and useful form of social and emotional support during difficult transition experiences. Furthermore, recent research on inclusion of moderators in online wellness groups (Huh et al. 2013) and the effect of individual choice in managing Facebook feeds (Bakshy et al. 2015) offer opportunities for the introduction of evidence-based information.

The fact that women widely used mobile apps for health information during pregnancy, but reported apps as unavailable or invaluable postpartum, highlights the need for the development of more mobile apps with postpartum content. Such apps should be free, deliver relevant content in sync with the child’s age, and give users the ability to link to external, validated websites for more detailed information on specific topics of interest. Given the high level of need expressed for information on breastfeeding, mobile apps that educate based on practical simulation, like a prototype app that teaches users how to get a good latch (Emrick et al. 2011), represent a possible area for future intervention to fulfill the information needs of mothers.

Additionally, our study suggests that engagement with current social media would extend the reach of existing trusted face-to-face sources that provide evidence-based health information through online communities. This will require understanding of page-rank strategies, using available methods to highlight authoritative knowledge (like Google AdWords), and increasing significant engagement with face-to-face clients. The mere existence of a Facebook page or website will not induce women to consult it. Sustained activity on social media is necessary to link face-to-face or online authoritative websites to women’s needs. A key aspect of successful social media engagement is community creation; therefore online activity must extend beyond information push. Furthermore, it is important for health providers, federal organizations and others seeking to improve maternal and infant health outcomes, to engage with emergent social media (Pinterest, Twitter, Tumblr, Snapchat, Instagram, etc.) to be ready for the next generation of mothers and their social media preferences.

### Limitations

This was an exploratory study with limited generalizability to the general population. The study site is a college town, which may have affected the education level among the sample. The recruitment of interviewees was restricted to clients of our community partners. This recruitment strategy provided important data to community partners, but limited our sample size. Finally, the wide inclusion criteria of having a child 48 months or younger could have led to recall bias among interviewees who were further into the postpartum period.

## Footnotes

1 In Indiana, this means women living in households with a monthly pretax income of $4134 or less for a family of four.
